# Supplementary material for: Discovering Native Ant Species with the Potential to Suppress Red Imported Fire Ants
Source: Insects. 2024 Jul 31;15(8):582. doi: 10.3390/insects15080582 (PMC11354274; doi:10.3390/insects15080582)
Supplement: Supplementary file 1 [file insects-15-00582-s001.zip › Table S1.pdf]

**Table S1.** Information about NCBI GenBank accession numbers of ant species in this study.

| Species | Accession numbers | Size (bp) |
|---|---|---|
| *Monomorium chinense* | OK090901 | 657 |
| *Nylanderia bourbonica* | OK090904 | 657 |
| *Iridomyrmex anceps* | OK090898 | 657 |
| *Tetramorium caespitum* | OK090908 | 657 |
| *Ochetellus glaber* | OK090902 | 657 |
| *Pheidole* sp.#7 | OK091181 | 657 |
| *Pheidole* sp.#9 | OK091182 | 657 |
| *Crematogaster* sp. | OK091174 | 657 |
